# Computational and experimental pathways to next-generation ultrawide-band-gap oxide semiconductors

**DOI:** 10.1186/s40580-026-00534-4

**Published:** 2026-02-04

**Authors:** Sieun Chae, Jongin Kim, Joshua R. Anderson, Sanghyun Hong, Yaser Mike Banad, Hanjong Paik

**Affiliations:** 1 School of Electrical Engineering and Computer Science, Oregon State University, Corvallis, OR 97331 USA; 2 Department of Physics, Oregon State University, Corvallis, OR 97331 USA; 3 School of Electrical and Computer Engineering, University of Oklahoma, Norman, OK 73019 USA; 4 Center for Quantum Research and Technology, University of Oklahoma, Norman, OK 73019 USA

**Keywords:** Ultrawide-band-gap (UWBG) oxide semiconductors, High-throughput computational screening, Density functional theory (DFT), Carrier mobility and dopability, Epitaxial thin films growth, Electronic transport properties

## Abstract

Ultrawide-band-gap (UWBG) oxide semiconductors are emerging as key platforms for next-generation energy-efficient, high-power, and high-frequency electronics. Further performance gains require the discovery of alternative UWBG systems with band gaps well above 4 eV, while offering intrinsically higher carrier mobility and controllable dopability. This paper highlights recent computational predictions and experimental advances toward such materials, with a focus on dopant activation, carrier density control, and phonon-limited mobility. Although computational screening has uncovered numerous promising candidates, most have yet to be realized as high-quality, single-crystalline thin films suitable for scalable devicefabrication. While epitaxial growth offers a unique platform to probe the intrinsic properties of these materials, the experimental realization is often limited by kinetic and processing constraints, such as nucleation dynamics and precise control of growth parameters. This gap often leads to significant deviations from the theoretical ground-state electrical transport properties. We examine the origins of these discrepancies and highlight integrated approaches, combining high-throughput computation with advanced epitaxial synthesis, to accelerate the development of next-generation UWBG oxide semiconductors.

**Graphical abstract:**

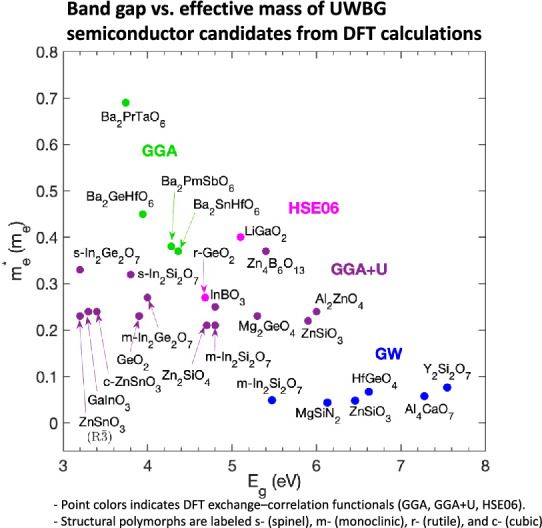

**Supplementary Information:**

The online version contains supplementary material available at 10.1186/s40580-026-00534-4.

## Introduction

Ultrawide-band-gap (UWBG) semiconductors are generally regarded as materials with a band gap (*E*_*g*_) exceeding that of GaN (3.4 eV), typically > 4.0 eV. Owing to their wide band gap and its corresponding high breakdown field, a UWBG semiconductor enables device operation at high voltages, frequencies, and temperatures. These characteristics make them attractive for applications such as power electronics, RF circuits, deep-UV optoelectronics, and electronics in harsh environments. Recently, the rising power demand in artificial intelligence (AI) technology further motivates the use of UWBG semiconductor-based power electronics, as they can deliver higher power while minimizing energy loss. Specifically, the ability to operate at high voltage (*V*) of UWBG semiconductors reduces the amount of current (*I*) required to deliver a given power, thereby improving the energy efficiency of power conversion, since power loss (*P*) is proportional to *I*^2^.

Despite their compelling advantages, the widespread adoption of UWBG semiconductors remains constrained by material-specific challenges. The most extensively studied UWBG semiconductor candidates include $\beta$-Ga_2_O_3_ (4.5 eV), diamond (5.5 eV), AlGaN (up to 6.0 eV), and *c*-BN (6.4 eV) [1]. Diamond and AlGaN are limited by their high cost and scarcity of lattice-matched substrates. $\beta$-Ga_2_O_3_ suffers from its intrinsically low thermal conductivity (10–27 W/m K), raising reliability concerns for high-power operation [2], while diamond, despite its exceptional thermal conductivity (~ 2200 W/m K), is hindered by poor dopability. In addition, *c*-BN remains difficult to synthesize and exhibits inefficient doping [3, 4]. These limitations urge researchers to explore alternative material systems. For future UWBG semiconductors to enable energy-efficient and reliable electronic devices, they must meet the requirements including (1) a sufficiently wide band gap, (2) reliable dopability, (3) high thermal conductivity, (4) high carrier mobility, and (5) the ability to produce high-quality thin films suitable for scalable electronic device fabrications.

Efforts to discover high-performance UWBG semiconductor materials are being advanced by both theoretical and experimental approaches. On the theoretical side, high-throughput computational searches—often aided by machine learning (ML) and materials informatics—narrow down the material space using descriptors readily available from a vast material database [5]. These screening processes are refined with first-principles calculations based on density functional theory (DFT) for accurate prediction of descriptors [6]. This method often involves rigorous computation that uses high-performance computing resources; however, it enables more accurate prediction of material properties based on the understanding of atomic-level origin, allowing theorists to design promising UWBG semiconductors with desired material properties.

Experimentally, material discovery has come from both the modification of known UWBG semiconductors [7–9] and synthesizing new compounds predicted by theory [10, 11]. Modification strategies include: (1) introduction of novel dopants to enhance transport properties [12], (2) alloying with chemically analogous compounds to tune band gaps or band alignments [7–9], and (3) exploring polymorphs or an atomically engineered structure, such as superlattices, to improve structural or electronic functionalities [13, 14]. At the same time, experimentalists have succeeded in synthesizing previously unexplored UWBG compounds that are predicted computationally, though this synthesis often faced challenges such as the phase competition, off-stoichiometric compounds, and miscibility gap/spinodal decomposition [15]. Even when the synthesis is successful, a significant discrepancy in material properties frequently arises between theoretically predicted and experimentally observed properties, as theoretical calculations typically represent the upper performance limit.

This review aims to highlight recent advances and the challenges in UWBG semiconductor discovery, from both theoretical and experimental perspectives. In contrast to prior materials-focused reviews, this article emphasizes the translation from prediction to realization by linking computational design with experimental epitaxy, mapping predicted candidates to their realization pathways, and identifying key bottlenecks that hinder validation. We first overview the computational methodologies and summarize the identified candidate materials. Next, we examine the experimental strategies to realize the theoretically predicted UWBG compounds and assess the extent to which theoretical predictions have been validated. Finally, we discuss synthesis-related challenges that contribute to the misalignment between theory and experiment and conclude with a future perspective on integrated research directions to accelerate material discovery from prediction to realization.

## High-throughput computational search

**Fig. 1:**
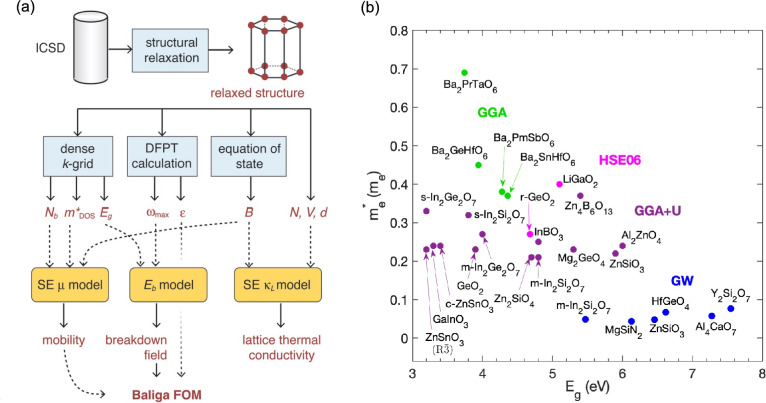
**a** Workflow for calculating the Baliga figure of merit (FOM) and lattice thermal conductivity ($\kappa_{L}$) using high-throughput accessible material parameters. Symbols: *N*_*b*_—band degeneracy; m^*^_DOS_—the density of state effective mass; *E*_*g*_—the band gap; $\omega_{max}$—the phonon cutoff frequency at ${\Gamma }$; $\varepsilon$—the dielectric constant; *B*—the bulk modulus; *N*– the number of atoms in the primitive cell; *V*—the primitive cell volume; *d*—the density; $\mu$—the mobility; *E*_*b*_—the breakdown field. SE stands for semi-empirical and DFPT for density functional perturbation theory. The figure is adapted in ref. [5]. **b** Band gap and effective mass of theoretically identified UWBG semiconductor candidates. The point color of each data point denotes the type of exchange–correlation functional used in the DFT band gap calculation. For In_2_Ge_2_O_7_, In_2_Si_2_O_7_, ZnSnO_3_, and GeO_2_ polymorphs, the label s-, m-, r-, c- indicate spinel, monoclinic, rutile, and cubic structure, respectively. Data sourced from refs [5, 18–21] and complete dataset provided in Table S1
- *Lattice thermal conductivity* Temperature (*T*)-dependent $\kappa_{L}$ for acoustic and optical phonon modes is semi-empirically modeled by fitting DFT data to experimental data:
  $$ \kappa_{L} = A_{1} \frac{{Mv_{s}^{y} }}{{T\gamma^{2} V^{z} n^{x} }} + A_{2} \frac{{v_{s} }}{{V^{z} }}\left( {1 - \frac{1}{{n^{2/3} }}} \right) $$
  where $M$ is the average atomic mass, $v_{s}$ is the speed of sound, $\gamma$ is the Grüneisen parameter, $V$ is the volume per atom, $n$ is the number of atoms in the primitive cell, and $A_{1}$, $A_{2}$, $x$, $y$, and $z$ are fitted parameters [17].

A high-throughput computational approach systematically explores a vast space of materials database to identify the most promising candidates through a hierarchical screening process. The workflow of the high-throughput search of UWBG semiconductors was first introduced by Gorai et al. [5] (Fig. 1a). Two descriptors are employed in screening materials: (1) the Baliga figure of merits (BFOM = $\varepsilon \cdot \mu \cdot E_{b}^{3}$ where $\varepsilon$ is dielectric constant, $\mu$ is carrier mobility, and $E_{b}$ is critical breakdown field)—which quantifies the power conversion efficiency of UWBG semiconductor devices, and (2) lattice thermal conductivity ($\kappa_{L}$)—which reflects the heat dissipation ability during device operation. While $\varepsilon$ can be directly obtained by density functional perturbation theory (DFPT) and is readily available in a material database, $\mu$, $E_{b}$, and $\kappa_{L}$ require extensive calculations that are challenging to perform in a high-throughput framework. To address this limitation, Gorai et al. developed a (semi)-empirical model and a ML-assisted approach to efficiently estimate these properties:

- *Mobility* The phonon-limited $\mu$ is modeled by $\mu = A_{0} B\left( {m^{*} } \right)^{t}$ where $A_{0}$ and *t* are fitted parameters, *B* is the bulk modulus, and $m^{*}$ is an effective mass. This model assumes that the scattering mechanism depends on the bulk modulus due to phonon-induced deformation, and the scattering rate depends on the electron density of states (DOS), which is related by $m^{*}$. *B* and $m^{*}$ are accessible parameters in a material database.
- *Breakdown field* $E_{b}$ is phenomenologically modeled by ML-training of DFT-computed $E_{b}$ for 82 insulators to relate with high-throughput accessible material parameters:
  $$ E_{b} = 24.442e^{0.315} \sqrt {E_{g} w_{max} } $$
  where $E_{g}$ is a band gap and $w_{max}$ is phonon cutoff frequency [16].

By applying these descriptors, the authors calculated the BFOM and $\kappa_{L}$ for 863 materials, including oxides, nitrides, carbides, sulfides, and borides. Materials exhibiting a BFOM greater than that of Si and $\kappa_{L}$ greater than 20 were identified as promising UWBG semiconductor candidates. The top seven candidates—HfGeO_4_, Y_2_Si_2_O_7_, MgSiN_2_, Al_4_CaO_7_, ZnSiO_3_, In_2_Si_2_O_7_, and HfSiO_4_—are highlighted with blue circles in Fig. 1b, with their band gaps and effective masses indicated.

In a follow-up study, the authors focused specifically on oxide candidates and implemented an additional screening procedure to more accurately evaluate the properties of materials by applying hybrid DFT calculations [18]. This paper explicitly considers the dopability of materials, which are initially screened through the branch point energy (EBP) method and subsequently predicted by using first-principles defect calculations. This approach revealed previously unexplored n-type semiconductors, including (III)_2_(IV)_2_O_7_ with pyrochlore or thortveitite structure, (III/II)_2_(II/IV)O_4_ with spinel or other trigonal structures, and (III)BO_3_. The 14 top candidates identified, along with their properties, are shown in Fig. 1b (purple).

Separately, Shen et al. applied a high-throughput method to discover novel UWBG double perovskites, A_2_BB’X_6_ [19]. From an initial set of 71,492 candidates—generated by combining 50 A-site cations, 64 B-site cations, and 4 X-site anions—thermodynamically stable materials were further screened based on the Goldschmidt tolerance factor. DFT data from a material database was then used to train ML models that predict band gap and stability based on composition. Applying these models, followed by DFT calculations, the study identified four UWBG double perovskites with an effective mass lighter than 0.7 (see Fig. 1b, light green).

## First-principles computational search

High-throughput screening method enables rapid survey a large number of material candidates, while first principles density functional theory (DFT) calculations offer accurate predictions of their intrinsic properties of materials. For UWBG semiconductors, key performance-determining properties include band gap, band alignment, defects and doping behavior, polaron formation, free carrier concentration controllability and mobility, and thermal conductivity.

Among these aforementioned properties, dopability is a critical factor distinguishing UWBG semiconductors from pure insulators. Unlike traditional semiconductors (e.g., Si) exhibiting high doping efficiency, doping becomes increasingly difficult as the band gap widens. This challenge arises because UWBG materials typically have deeply positioned valence bands or highly elevated conduction bands, placing the dopant energy level far from the band edges [22]. This resulted in a large dopant activation energy and suppressed doping efficiency. Furthermore, UWBG oxide semiconductors easily form the charged native-defects, such as vacancies or common impurity complexes, that compensate dopants generated free carriers, which pins the Fermi level deep within the band gap [23]. Consequently, evaluating the dopability through first-principles defect (formation energy) calculation is essential for determining whether a UWBG material can exhibit the true semiconducting behavior.

**Fig. 2:**
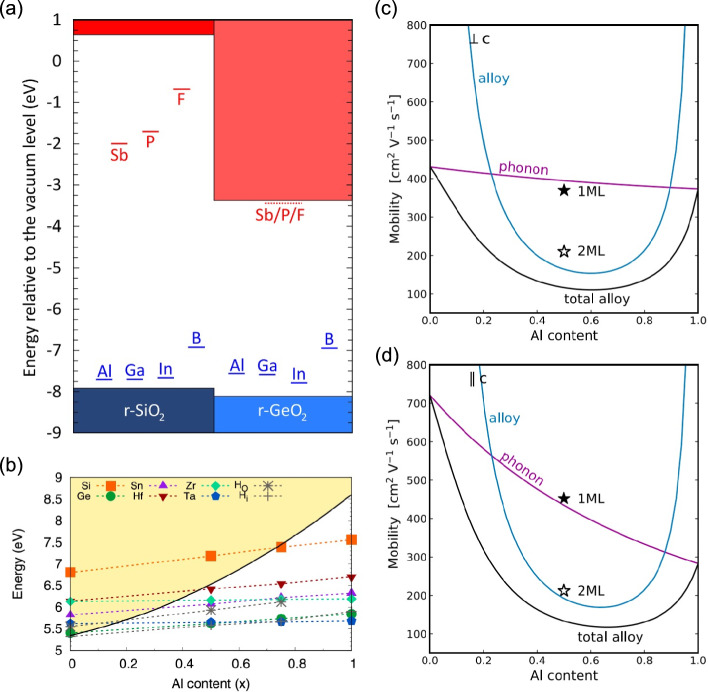
**a** First-principles band alignment of r-SiO_2_ and r-GeO_2_ and the transition levels of potential *n*-type (red) and *p*-type (blue) dopants. **b** Transition levels of various n-type dopants in corundum (Al_x_Ga_1-x_)_2_O_3_ alloy as a function of Al composition. The shaded yellow region indicates the conduction band minimum (CBM) energy level of (Al_x_Ga_1−x_)_2_O_3_ alloy referenced to the valence band maximum (VBM) energy level of Ga_2_O_3_. **c**, **d** First-principles calculation of electron mobility in AlN/GaN superlattices compared with Al_x_Ga_1−x_N random alloys: **c** in-plane (perpendicular to the c-axis, $\bot$c) mobility, and **d** out-of-plane (parallel to the c-axis, ${\|}$c) mobility. “1ML” and “2 M” denote superlattice with one- and two-monolayer periodicities, respectively. Figure **a** is adapted from ref. [26], figure **b** from ref. [28], and figure **c**, **d** from ref. [34]

In the modern framework of point-defect calculation, pioneered by Van de Walle and co-workers [24], the formation energy of charged and neutral point defects in a semiconductor are calculated as a function of the Fermi energy level under specified chemical potential conditions. In these calculations, the use of hybrid functional enables accurate prediction of band gaps, defect ionization energies, and equilibrium Fermi energy levels. In addition, by explicitly computing chemical potentials, one can identify the favorable material growth conditions that enhance doping efficiency. In this methodology, it allows prediction of polarons formation by comparing the energies of a localized polaron—accompanied by lattice distortion—with those of a delocalized state [25]. Using this methodology, theorists have identified numerous UWBG materials with promising doping characteristics. Rutile GeO_2_, with a band gap of 4.67 eV, is predicted to be an ambipolarly dopable semiconductor with Sb_Ge_, As_Ge_, P_Ge_, and F_O_ acting as donors, while Al_Ge_, Ga_Ge_, and In_Ge_ serving as acceptors (Fig. 2a) [20, 26]. Similarly, Lyons et al. [26] predicted that rutile SiO_2_ is a promising p-type dopable semiconductor despite its large band gap of 8.57 eV (Fig. 2a). Unlike many other wide-band-gap oxides, self-trapped holes in rutile SiO_2_ are unstable, facilitating p-type doping. The favorable *p*-type characteristics of both r-GeO_2_ and r-SiO_2_ arise from the densely packed oxygen sublattices of rutile compounds, which leads to a light hole effective mass. In addition, the Li-Ga-O system has emerged as a promising *n*-type UWBG (> 5 eV) semiconductor. LiGaO_2_ is a wurtzite-structure derived crystal with a band gap of 5.1 eV, which is predicted to be *n*-type dopable via substitution of Ga with Si, Ge, and Sn [21, 27]. Furthermore, Wickramaratne et al. [28] demonstrated that corundum-structured (Al_x_Ga_1−x_)_2_O_3_ remains n-type dopable: Si acts as a shallow donor on the Ga site for Al concentration up to 72%, corresponding to a band gap of 7.5 eV (Fig. 2b).

For the predicted dopable UWBG materials, first-principles calculations of carrier mobility have also been reported. Mobility can be obtained by combining band structure calculation—using many-body perturbation theory or hybrid functional DFT—with first principles calculations of electron scattering rates from phonons, defects, and alloy disorder, and then iteratively solving the Boltzmann transport equation (BTE) [6, 29]. The more efficient formalism for computing carrier scattering rates is also described in ref. [30]. It provides reasonable accuracy of results by using low-cost ab initio inputs. First-principles approaches enable the calculation of mobility as a function of temperature, carrier concentration, strain, or composition, while elucidating the contribution of each scattering mechanism. This framework not only allows mobility prediction prior to experimental measurements but also offers guidance for the rational design of high-mobility materials.

Using this approach, Bushick et al. predicted that phonon-limited electron mobility of r-GeO_2_ is 244 cm^2^ V^−1^ s^−1^ perpendicular to the *c*-axis ($\bot$*c*) and 377 cm^2^ V^−1^ s^−1^ parallel to the *c*-axis (${\|}$*c*) at 300 K, while hole mobility is 27 cm^2^ V^−1^ s^−1^ ($\bot$*c*) and 29 cm^2^ V^−1^ s^−1^ (${\|}$c). These predictions, made prior to the experiment, highlight the potential of r-GeO_2_ for power electronics as well as CMOS applications [31]. Duan et al. further investigated the mobility of ordered $\beta$-(Al_x_Ga_1−x_)_2_O_3_ alloys with varying Al composition, showing that the electron mobility decreases with the increase of Al fraction due to the enhanced polar optical phonon (POP) scattering [32]. In parallel, Pant et al. calculated alloy-disorder-limited electron mobility in Al_x_Ga_1−x_N from first principles where the scattering lifetime is evaluated by unfolding the band structure from the supercell basis to the primitive-cell basis (Fig. 2c, d). In 0.25 < x < 0.85, the mobility is limited by alloy scattering, which reaches a minimum at the most disordered composition of 0.5 < x < 0.6, revealing that alloy scattering drastically reduces electron mobility by introducing statistical disorder in crystal momentum [33]. On the other hand, AlN/GaN superlattices (i.e., a periodic structure of AlN and GaN layers) can effectively enhance mobility by eliminating alloy-disorder scattering (Fig. 2c, d) without reducing a band gap [34]. Superlattices with monolayer periodicity (denoted by 1ML in Fig. 2c, d) exhibits mobility close to the phonon-limited mobility, leading to > 3 times higher mobility compared to Al_0.5_Ga_0.5_N alloy. The mobility of superlattices with two-monolayer periodicities (2 ML) is smaller than 1ML superlattice due to the increased number of phonon modes that scatter electrons but is 1.6 times higher than Al_0.5_Ga_0.5_N alloy due to the absence of alloy scattering.

## Experimental material discovery

Despite extensive theoretical predictions, only a limited number of UWBG oxide materials have been experimentally synthesized and characterized. Those materials include $\alpha$- and $\beta$-phases of (Al_x_Ga_1−x_)_2_O_3_ alloys [7, 35], r-GeO_2_ as well as r-(Ge_x_Sn_1−x_)O_2_ alloys [10, 36], Ga-related ternary compounds such as MgGa_2_O_4_, ZnGa_2_O_4_, and LiGa_5_O_8_, [11, 37] and perovskite stannates [38–40].

**Fig. 3:**
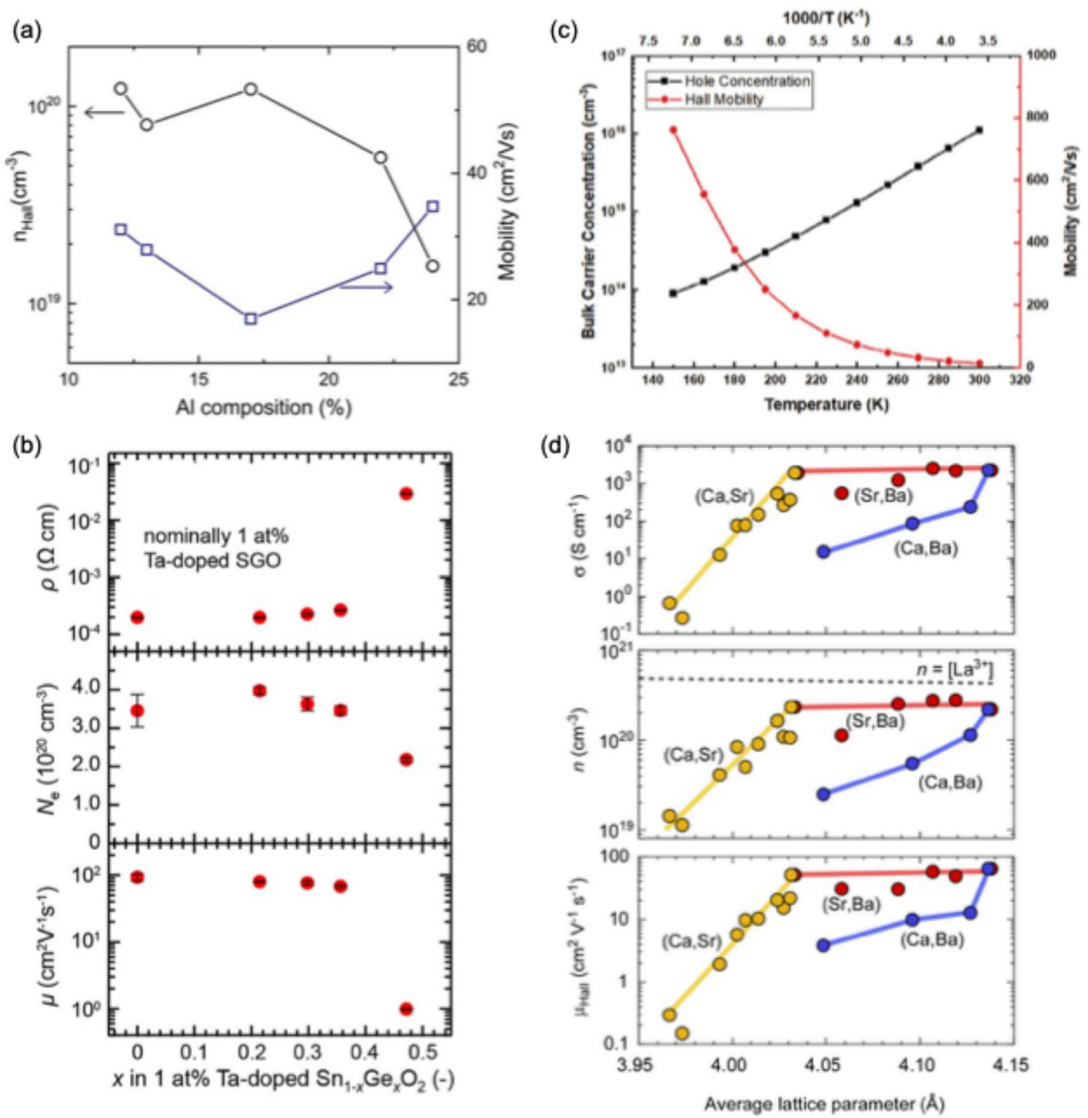
Experimental demonstration of novel UWBG semiconductor compounds. **a** Hall carrier concentration (n_Hall_) and electron mobility of Si-doped $\beta$-(Al_x_Ga_1−x_)_2_O_3_ as a function of Al composition (x). **b** Electrical resistivity ($\rho$), carrier concentration (*N*_*e*_), and electron mobility ($\mu$) of Ta-doped r-(Ge_x_Sn_1−x_)O_2_ thin films as a function of Ge content (x). **c** Temperature dependent carrier concentration and hole mobility of p-type LiGa_5_O_8_. **d** Electrical conductivity ($\sigma$), carrier concentration (*n*), and electron mobility ($\mu$_Hall_) of La-doped Ca_1−x_Sr_x_SnO_3_, Sr_1−x_Ba_x_SnO_3_, and Ca_1-x_Ba_x_SnO_3_ solid solution thin films as a function of average lattice parameter. Figure **a** is adapted from ref. [43], **b** from ref. [36], **c** from ref. [11], and **d** from ref. [48]

First, alloying has been an effective strategy to tune the properties of UWBG oxide semiconductors. For example, $\alpha$-(Al_x_Ga_1−x_)_2_O_3_ thin films, across the full composition range of *x,* have been synthesized on sapphire substrates by molecular beam epitaxy (MBE) [7, 41] and metalorganic chemical vapor deposition (MOCVD) [42], achieving band gaps from 5.4 eV to 8.8 eV. However, high electrical conductivity and effective doping in $\alpha$-(Al_x_Ga_1−x_)_2_O_3_ films remain challenging. In contrast, highly electrical conductive (> 610 S cm^−1^) Si doped $\beta$-(Al_x_Ga_1−x_)_2_O_3_ thin films are reported for 0.12 < *x* < 0.24, where films are coherently strained on a $\beta$-Ga_2_O_3_ substrate [43]. The electrical characteristics of $\beta$-(Al_x_Ga_1−x_)_2_O_3_ thin films determined by Hall effect measurement are shown in Fig. 3a.

Second, rutile-(Ge_x_Sn_1-x_)O_2_ alloys offer a tunable band gap between 3.7 and 4.7 eV. r-(Ge_x_Sn_1−x_)O_2_ thin films spanning the full composition *x*-range have been grown on TiO_2_ (001) and Al_2_O_3_ (10–10) substrates by using mist CVD [9] and pulsed laser deposition (PLD) techniques [36]. Low-Ge-content r-(Ge_x_Sn_1−x_)O_2_ thin films (*x* < 0.36) grown on Al_2_O_3_ substrates are *n*-type dopable with Ta^5+^, exhibiting high carrier density (over 3 × 10^20^ cm^−3^) and Hall mobility of 70 cm^2^ V^−1^ s^−1^ (Fig. 3b) [36]. r-(Ge_0.49_Sn_0.51_)O_2_ films have been further used to fabricate lateral Schottky barrier diodes, demonstrating their potential as a practical semiconductor [44]. High-Ge content r-(Ge_x_Sn_1-x_)O_2_ thin films, however, still require further optimization to achieve higher electrical conductivity.

Third, spinel Ga-based ternary oxides have been explored as alternative UWBG oxide semiconductors. Inverse spinel MgGa_2_O_4_ (E_g_ = 4.9 eV) bulk single crystals exhibit *n*-type conductivity but suffer from low mobility [45]. Spinel ZnGa_2_O_4_ (E_g_ = 4.6 eV) has shown high mobilities (bulk Hall mobility = 107 cm^2^ V^−1^ s^−1^; field-effect mobility = 5.4 cm^2^ V^−1^ s^−1^) [46, 47], yet challenges in carrier concentration control and limited thermal conductivity (22.1 W m^−1^ K^−1^), which restrict its application in power electronic devices [46]. Recently, Zhang et al. demonstrated successful growth of spinel LiGa_5_O_8_ epitaxial thin films utilizing the mist-CVD growth technique. It displays robust *p*-type conductivity at room temperature with a broad range of hole concentration from 10^15^ cm^−3^ to 10^18^ cm^−3^ [11]. Further optimizing the Li/Ga ratio could enhance control over hole carrier concentration, making LiGa_5_O_8_ the widest band gap (E_g_ = 5.36 eV) *p*-type oxides reported to date. Figure 3c presents temperature-dependent Hall effect measurement of a selected LiGa_5_O_8_ sample.

Fourth, La-doped perovskite stannate (ASnO_3_, A = Ba, Sr, and Ca) is another promising class of UWBG oxide semiconductors discovered by experimentalists where room temperature electron transport properties of La-doped Ca_1-x_Sr_x_SnO_3_, Sr_1-x_Ba_x_SnO_3_, and Ca_1-x_Ba_x_SnO_3_ solid solution films as a function of average lattice parameter is shown in Fig. 3d. The band gap increases (from 3.2 to 4.4 eV) with decreasing ionic radius of the A-site ion (from Ca^2+^ to Ba^2+^), while electrical conductivity decreases [40, 48]. Stannates with E_g_ > 4.0 eV include SrSnO_3_ (4.1 eV), CaSnO_3_ (4.4 eV), and (Ca_1−x_Sr_x_)SnO_3_ alloys (4.1–4.4 eV) [48]. SrSnO_3_ thin films reported mobility up to 70 cm^2^ V^−1^ s^−1^ at *n* = 1.3 × 10^20^ cm^−3^, while CaSnO_3_ thin films mobility show 42 cm^2^ V^−1^ s^−1^ at *n* = 3.3 × 10^19^ cm^−3^ [38, 39]. MESFET based on La-doped CaSnO_3_ demonstrate low off-state current (< 2 × 10^–5^ mA/mm) at a drain-source voltage of 100 V and on–off ratios > 10^6^, highlighting their potential for high-voltage electronics [39].

## The gap between theory and experiment

The extensive computational search identified numerous promising UWBG semiconductor compounds (Fig. 1b). Of the 26 top candidates predicted by theory; a literature survey revealed that 22 materials have been experimentally synthesized (Table S1). However, most reports involve bulk, powder, or polycrystalline thin films, with the synthesis of single-crystalline thin films is achieved only for 4 materials: MgSiN_2_, ZnSnO_3_, r-GeO_2_, and LiGaO_2_ (Table S1) [15, 49–51]. Moreover, experimental demonstration of electronic transport or effective doping has been rarely reported [52]. In this section, we analyze key challenges that hinder the translation of theoretical prediction into experimental realization.

**Fig. 4:**
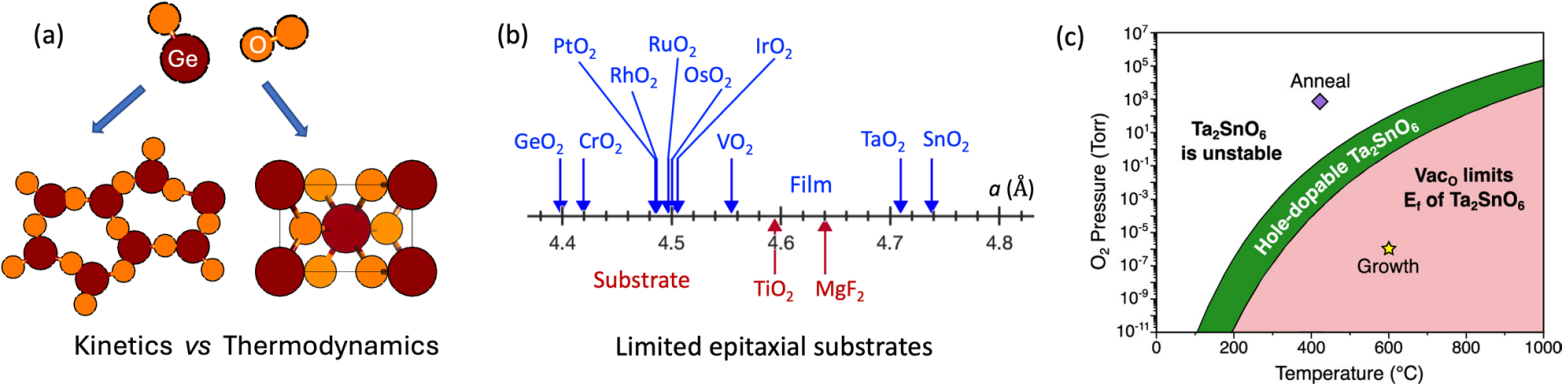
Challenges of growing UWBG semiconductors identified by theory: **a** mismatch between kinetically favorable compounds and thermodynamically favorable compounds, **b** limited availability of epitaxial substrates with small lattice misfit, **c** limited window of chemical potential to synthesize materials with activated carriers. **c** Is adapted from ref. [58]

A primary challenge lies in thin films growth of UWBG semiconductors. While theoretically proposed compounds may be thermodynamically stable, they are not always kinetically favorable for synthesis. Conventional computational search methods often use the energy above the convex hull (E_hull_)—the formation energy difference between a compound and the thermodynamic convex hull—as a descriptor for synthesizable materials. This assumes that synthesizability can be predicted by the relative stability of a compound compared to its ground state. However, E_hull_ is a DFT-calculated quantity assuming at T = 0 K. In practice, experimentalists rely on a phase diagram to discover the growth conditions of a desired phase. While this method is effective for bulk crystal growth, thin film synthesis typically involves rapid, non-equilibrium processes dominated by kinetics, which are not captured by thermodynamic descriptors alone.

One example is r-GeO_2_ thin film growth. Although the rutile phase is the most stable form of GeO_2_ up to 1030 °C [53], GeO_2_ is also a well-known glass former, and the rutile phase can only be synthesized under a restricted growth condition [15]. In addition, due to the high volatility of GeO_2_, film stoichiometry strongly depends on the kinetic path of precursors (Fig. 4a). For example, in molecular beam epitaxy of GeO_2_, the use of a suboxide precursor allows for the rutile phase stabilization via the simple reaction of GeO (g) + ½ O_2_ (g) → GeO_2_ [54, 55] while the use of pure Ge precursor complicates the oxidation steps (i.e., Ge (g) + ½ O_2_ (g) → GeO (g), GeO (g) + ½ O_2_ → GeO_2_ (s)) which further limits the growth windows to achieve the targeted crystalline phase and stoichiometry [54]. However, film kinetics is not considered in the computational survey. As a result, experimentalists often rely on lengthy trial-and-error periods to synthesize materials.

Another growth challenge arises when a compound includes an element that is hazardous or difficult to process. For example, among the UWBG semiconductor double perovskites predicted by theorists, Ba_2_PmSbO_6_ contains a radioactive element (promethium). Lithium is also an element known for high reactivity and high volatility, which leads to growth challenges associated with Li loss [56]. Although K. Zhang reported the growth of *p*-type LiGa_5_O_8_ thin films using the mist-CVD technique, difficulty in controlling film stoichiometry led to variations in hole concentration and made it difficult to control *p*-type conductivity [11].

In addition, the lack of suitable lattice-matched substrates or seed crystals hinders the experimental realization of new UWBG compounds (Fig. 4b). Conventional computational search method focuses on single-crystal compounds due to the lower computational cost of modeling them. However, experimental synthesis of high-quality single crystals is challenging. Single-crystalline thin films are grown by epitaxial techniques, where lattice-matched single crystalline substrates or seed crystals are used as a template for crystal growth. While suitable substrates are available for high-symmetry crystals such as perovskite, materials having a low-symmetry crystal structure are difficult to synthesize due to the lack of isostructural substrates. Among the materials screened in Fig. 1b, 35% have either monoclinic or orthorhombic structure that lack suitable epitaxial substrates (Fig. 4b).

Furthermore, even if the synthesis is achieved, challenges associated with defect control can lead to significant discrepancies in material properties between theoretical prediction and experimental characterization. For example, epitaxial strain can induce substantial modifications in semiconductor properties. Specifically, epitaxial strain imposed by lattice and interfacial mismatch can alter the electronic structure, including band gap and effective mass [57]. Strain relaxation is governed by interface-driven factors, for instance, lattice mismatch, symmetry mismatch, anion sublattice mismatch (e.g., oxygen coordination across the interface), and polarity-induced electrostatic boundary conditions. Relaxation may proceed through elastic distortion, point defect formation, or extended defects such as misfit and threading dislocations. Those extended defects introduce structural scattering centers. Their cores and associated strain and electrostatic fields can be charged and act as preferential sites for dopant segregation or compensation, thereby enhancing ionized impurity scattering. At high dislocation densities, the combined effects of Coulomb scattering and structural disorder lead to pronounced carrier mobility degradation [60, 61]. In addition, thin films of multi-cation compounds involve cation disorder and local off-stoichiometry due to the entropy effect, which introduces an additional scattering mechanism that may be neglected in theory [33]. Consequently, theoretical predictions often represent the upper bounds of semiconductor properties, whereas experimentally measured properties are typically lower.

Defect control is particularly important for optimizing the effect of doping. In UWBG semiconductors, native point defects such as vacancies or interstitials often provide carriers that compensate free carriers generated by dopants. Therefore, extreme care is needed to control the formation of native defects to maximize doping efficiency. In theory, a chemical potential diagram is used to predict the chemical potential windows that enable doping of semiconductors [58]. However, if this chemical potential window is too narrow, achieving the desired doping properties becomes challenging. For instance, *p*-type conductivity of Ta_2_SnO_6_ is predicted by theory; however, the limited chemical potential range for acceptor-type doping has hindered the experimental demonstration of *p*-type conduction of Ta_2_SnO_6_ (Fig. 4c). Under O-rich growth condition, impurity phases such as SnO_2_ or Ta_2_O_5_ are likely to form, rendering Ta_2_SnO_6_ unstable, whereas O-poor growth condition generates O vacancies that eliminate hole carriers as described in Fig. 4c [58]. This constraint becomes increasingly significant as the number of component species increases.

Lastly, interface effects can impede accurate electrical measurement of UWBG semiconductors, resulting in substantial discrepancies of material properties between theoretical prediction and experimental observation. For example, the substrate band gap imposes an additional constraint for growing a UWBG semiconductor thin film. To confine carriers in the semiconductor layer, a substrate with an even wider band gap is desired. Substrates with a narrower band gap can provide carriers, complicating the characterization of intrinsic electrical properties. For instance, although TiO_2_ is a commercial, isostructural substrate for r-GeO_2_, its narrower band gap (3.2 eV) as well as the presence of native free carriers hinder accurate characterization of r-GeO_2_ transport properties [59]. Instead, Al_2_O_3_ is used as an alternative substrate despite its large lattice mismatch and symmetry mismatch with r-GeO_2_. Additionally, many UWBG semiconductors exhibit high contact resistance due to the lack of suitable ohmic contact materials, which further complicates electrical characterization.

## Concluding remarks and outlook: AI-assisted design and synthesis of next-generation UWBG semiconductors

The development of ultra–wide-bandgap (UWBG) semiconductors is entering a new phase, where the physical theory, data-driven computation, and material synthesis converge to accelerate materials discovery and device development. Nevertheless, the practical impact of this convergence remains limited, primarily due to the intrinsic synthesis complexity and challenging defect physics of UWBG semiconductors. Historical experience from GaN research provides an important lesson: Even when theory predicts favorable dopability, this alone is insufficient to guarantee practical doping success. Achieving reliable *n*-type and* p*-type doping required decades of experimental refinement to overcome challenges, such as defect compensation, hydrogen passivation, dislocation-mediated scattering, and growth kinetic limitation.

Importantly, UWBG oxides face even more challenges, including strong polaron localization, deep defect levels, limited dopant solubility, and extreme sensitivity to oxygen chemical potential. Consequently, many UWBG oxide compounds appear promising in theory but remain experimentally inaccessible or irreproducible due to narrow kinetic growth windows and metastable phase competition. Precise control of stoichiometry, phase stability selection, dopant selection and activation, and interface/surface quality, often governed by lowest-energy surface configurations, remains a central challenge in UWBG oxide thin-film growth. These limitations highlight the need for *critically integrated approaches* that combine theory, computation, synthesis, together with AI-assisted strategies to enable *inverse design*, *defect engineering*, and *adaptive growth control* [63, 65, 69, 74, 76].

I. AI-assisted design

1. *Interpretable, physics-grounded prediction* Data-driven models now enable exploration of vast chemical and structural spaces. Those offer powerful generative and predictive capabilities for key semiconductor properties such as band gap, dielectric strength, dopability, and carrier mobility. However, for experimentalists, a major limitation remains; it is often unclear why a model makes a given prediction. To extract meaningful physical insight, future efforts must emphasize interpretable ML frameworks that explicitly connect predictions to underlying chemistry and physics. These relevant descriptors include structural bonding motifs, coordination environments, atomic orbital character, and defect energetics that directly influence the electronic behavior of UWBG oxides.
  Promising approaches include physics-aligned ML frameworks, such as **Physics-Informed Neural Networks** (**PINNs**) [62, 63], which are designed to incorporate physical laws, often expressed through differential equations, directly into the learning process. While PINNs have been extensively explored in general physics problems and offer more interpretable, physically grounded solutions, their applicability to complex material system (e.g., complex oxides) remains largely unexplored.
  In this context, key open questions include: (i) how to define relevant physical constraints for complex material systems, (ii) how to incorporate multiscale phenomena, and (iii) how to ensure that interpretability does not compromise predictive power. Addressing these challenges will be critical for developing ML models that not only accurately predict UWBG oxide semiconductor properties but also provide clear physical insight into the mechanisms governing their behavior, thereby offering practical experimental guidance on why certain compositions or structures succeed or fail.
  Recent studies have begun to demonstrate the feasibility of this approach in UWBG oxides. For example, ML models have been used to predict the Fermi level position in ultrawide-bandgap Ga_2_O_x_ thin films based on experimentally accessible processing parameters, enabling accelerated optimization of electrical properties relevant to device applications [64].
2. *Inverse design* High-throughput screening and first-principles defect calculations summarized in this review identify numerous promising UWBG oxides. However, only a small fraction of these materials has been realized as high-quality epitaxial films because synthesis remains constrained by growth kinetics, phase composition, and defect control.
  Inverse-design and Generative ML models offer an alternative path [65–67]. These model can propose candidate chemistries directly from target figures of merit (e.g., band gap, dielectric strength, mobility) after training on large databases of computed and measured properties.
  When combined with defect-aware descriptors (e.g., dopant activation energies or polaron formation energies) and process-relevant quantities (e.g., formation energy or decomposition enthalpy under growth conditions), these approaches could enable targeted exploration of chemical space for UWBG oxides that *simultaneously* optimize electronic performance and realistic synthesizability [68, 69]. Unlike conventional forward-screening strategies, this inverse design approach reframes materials discovery as property-driven-structure design process, which naturally aligning with the “prediction-to-realization workflow” required for experimentally viable UWBG oxide semiconductors [70].

II. AI-assisted synthesis: from target properties to real materials

Synthesis remains the dominant bottleneck for many UWBG oxides. Numerous theoretically predicted phases are metastable, highly defect-sensitive, or accessible only within narrow kinetic and processing windows. AI/ML tools offer several complementary strategies to address these synthesis challenges.

1. *Synthesizability prediction* The thermodynamic energy above the convex hull (E_hull_) is an imperfect indicator for synthesizability. Several compounds such as rutile GeO_2_ and Li-containing oxides, illustrate this limitation. Although thermodynamically stable, these materials can be difficult or even impossible to grow due to kinetic barriers, precursor volatility, or competition from secondary phases.
  Data-driven synthesizability models provide a more reliable alternative. Models trained on database such as International Crystal Structure Database (ICSD) and Materials Project–often incorporating both successful and failed synthesis attempts–can significantly outperform formation-energy-based criteria by large margins. In addition, these approaches help prioritize which UWBG oxides are most likely to be experimentally accessible.
  Recent frameworks extend this concept by integrating ML models into crystal-structure prediction and reaction-path planning. This enables synthesizability-driven structure searches and even suggestions of likely precursors and reaction conditions for a target phase. Representative examples include SynthNN—a deep neural network-based synthesizability model- and SyntheFormer, which combines Fourier-transformed crystal representations with hierarchical feature extraction, Random Forest feature selection, and a compact multilayer perceptron classifier [71–73].
  Incorporating such tools into UWBG discovery pipelines can help experimentalists focus limited synthesis effort on compounds and polymorphs that are both functionally promising and realistically accessible. However, device-relevant thin films, additional development is required. In particular, models trained on known growth successes and failures with semiconductor processing techniques such as physical vapor deposition methods (MBE, PLD, sputtering) or chemical deposition (CVD, ALD) are critically needed [74]. Ultimately, reliable synthesis prediction must go beyond simple thermodynamic metrics. It should explicitly incorporate kinetic effects, oxygen activity, precursor volatility, and phase competition–factors that dominate real growth processes but are often overlooked by equilibrium-based descriptors.
2. *Adaptive, real-time growth* Thin-film growth of UWBG semiconductors involves a high-dimensional parameter space including substrate choice and orientation, cation fluxes, oxygen activity, substrate temperature profile, and post-growth annealing conditions. These parameters strongly influence phase stability, defect chemistry, and transport behavior.
  By integrating ML-based tools–such as Bayesian optimization or active learning–with in situ diagnostics including Reflection High-Energy Electron Diffraction (RHEED), plume spectroscopy, and optical emission spectroscopy, the growth parameters can be dynamically adjusted during deposition to enable real-time process control [75].
  When coupled with automated synthesis platforms, such closed-loop systems can optimize processing conditions orders of magnitude faster than manual exploration. They can also learn “recipe maps” that approximate experimental phase diagrams, enabling stabilization of metastable phases and optimization of dopant incorporation with minimal manual intervention. However, current implementations remain limited by experimental noise, limited throughput, and poor reproducibility across tools and labs [74, 75].
  Related progress has been demonstrated in MBE, where ML models trained on real-time RHEED data have enabled dynamic prediction of surface morphology and adaptive control of growth conditions during deposition [76]. ML-assisted analysis of RHEED patterns has also been shown to improve interpretation of growth modes and crystallinity during thin-film deposition, providing a data-driven route to quantify epitaxial quality and growth kinetics [77]. Similar ML-assisted closed-loop strategies are also emerging in PLD, sputtering, and CVD, where plume, plasma, and optical diagnostics–such as Low Angle X-ray Spectroscopy (LAXS), or Ion Energy Spectroscopy (IES)–enable adaptive control of growth conditions and film quality [78, 79].
3. *Defect and interface engineering* ML models for charged defect formation energies and migration barriers are emerging as powerful surrogates for first-principles defect calculations, enabling rapid screening of dopants and compensating defects across thousands of oxides while bypassing thousands of supercell calculations. In UWBG research, such tools can be employed to guide dopants selection and control chemical potentials to maximize doping efficiency, with direct relevance to experimental growth conditions. Similar ML frameworks can also be applied to interface engineering including prediction of band alignment and contact resistance [80, 81]. This facilitates rational selection of substrates and metallization schemes that preserve intrinsic charge transport properties in UWBG films.

III. Integration: closing the loop between design and synthesis

The full value of AI/ML emerges when design and synthesis tools are tightly coupled and operate synergistically. “*Predictive models*” generate candidates that satisfy both functional and stability criteria; while “*synthesis models*” filter these compounds based on realistic growth windows. Experimental outcomes then feedback to refine both defect and property models.

Through multiple iterations, this closed-loop workflow builds a unified knowledge framework that links predicted materials to experimentally accessible phases, dopant behavior, and interface characteristics.

Beyond property prediction, closed-loop AI-guided synthesis has now been experimentally demonstrated in thin-film deposition. For example, autonomous pulsed laser deposition systems and self-driving sputtering systems that combine in-situ spectroscopy with ML optimization have achieved rapid convergence on optimal growth conditions, illustrating a practical route toward data-driven control of complex oxide growth [82, 83].

IV. Breakthrough potential

AI/ML–assisted frameworks promise several transformative capabilities, including:

- Predicting dopability and defect compensation *prior to synthesis*
- Rapid exploration of multi-cation or high-entropy oxide chemical spaces
- Self-correcting growth guided by real-time in-situ feedback
- Rational engineering of dopant activation pathways
- Predictive control of interfaces and contacts for reliable electronic properties

Recent studies already demonstrate key elements of this vision, including ML–assisted prediction of electronic properties in ultrawide-bandgap Ga_2_O_x_ thin films, autonomous optimization of pulsed laser deposition growth conditions, and real-time MBE growth control using RHEED-informed ML models [64]. These breakthroughs directly address the challenges that historically delayed *p*-type activation in GaN, enabling faster and more systematic pathways toward functional UWBG oxides. Collectively, these studies demonstrate that AI/ML-assisted prediction, closed-loop synthesis, and in-situ growth control are already being realized in thin-film systems. However, their systematic application to UWBG oxide epitaxy, particularly for defect control, dopant activation, and interface engineering, remains an important and timely opportunity for future research.

V. Overall perspective

AI/ML does not replace experimental intuition; rather, it amplifies human creativity by strategically directing effort toward the most promising compositions, growth conditions, and defect-management strategies. By integrating interpretable design models, synthesizability prediction, adaptive growth optimization, and rigorous experimental validation, the community can dramatically accelerate the translation of predicted oxides into device-ready thin films.

This integrated, data-driven, and experimentally anchored approach establishes a new materials discovery framework, positioning the field to systematically discover, synthesize, and optimize next-generation UWBG oxide semiconductors at a pace previously unattainable. In doing so, it opens transformative opportunities for deep-ultraviolet optoelectronics and devices operating in extreme environments. Ultimately, AI-guided synthesis can be viewed as a *powerful complement*, not a replacement, to deep physical understanding and experimental intuition.

## Supplementary Information

Supplementary Material 1

## Data Availability

No new datasets were generated or analyzed during the current study. Figures included in this review were redrawn or modified from previously published sources, which are appropriately cited.
