# Supplementary material for: Computational and experimental pathways to next-generation ultrawide-band-gap oxide semiconductors
Source: Nano Converg. 2026 Feb 4;13:5. doi: 10.1186/s40580-026-00534-4 (PMC12873060; doi:10.1186/s40580-026-00534-4)
Supplement: Supplementary file 1 — Supplementary Material 1 [file 40580_2026_534_MOESM1_ESM.pdf]

Table I. UWBG semiconductors discovered identified by theory and progress of material synthesis. SC no., E_g, method for E_g, $m_{e/h}$, and $\mu_{e/h}$ indicate space group, calculated band gap, type of functional used to calculate band gap within density functional theory, calculated electron/hole effective mass, and calculated electron/hole mobility, respectively. The theory data is from refs.[1–5]

Materials	Crystal structure	SG no.	E_g (eV)	Method for E_g	Carrier Type	$m_{e/h}$ (m_e)	$\mu_{e/h}$(cm^2 V^{-1} s^{-1})	Type of sample for synthesized material	Ref.
$HfGeO_4$	Orthorhombic	88	6.62	GW	*n*	0.067	-	Bulk	[6]
$Y_2Si_2O_7$	Monoclinic	12	7.55		*n*	0.077	-	Bulk	[7]
$MgSiN_2$	Orthorhombic	33	6.13		*n*	0.044	-	Thin film (epitaxy)	[8]
Al_4CaO_7	Monoclinic	15	7.28		*n*	0.058	-	Bulk	[9]
$ZnSiO_3$	Rhombohedral	148	6.46		*n*	0.048	-	Bulk	[10]
$In_2Si_2O_7$	Monoclinic	12	5.47		*n*	0.049	-	Bulk	[11]
$HfSiO_4$	Tetragonal	141	8.34		*n*	4.053	-	Bulk	[12]
Al_2ZnO_4	Spinel	227	6	GGA+U	*n*	0.24	1284	Powder	[13]
GeO_2	Orthorhombic	58	3.9		*n*	0.23	1513	Bulk	[14]
$GaInO_3$	Orthorhombic	194	3.3		*n*	0.24	1235	Powder	[15]
$In_2Ge_2O_7$	Spinel	227	3.2		*n*	0.33	616	Bulk	[16]
$In_2Ge_2O_7$	Monoclinic	12	4		*n*	0.27	691	Thin film (poly-crystalline)	[17]
$In_2Si_2O_7$	Spinel	227	3.8		*n*	0.32	811	Bulk	[18]
$InBO_3$	Rhombohedral	167	4.8		*n*	0.25	959	Thin film (poly-crystalline)	[19]
Mg_2GeO_4	Spinel	227	5.3		*n*	0.23	1293	Bulk	[20]
Zn_2SiO_4	Rhombohedral	122	4.7		*n*	0.21	1335	Thin film (poly-crystalline)	[21]
$ZnSnO_3$	Rhombohedral	148	3.4		*n*	0.24	953	Powder	[22]
$ZnSnO_3$	Rhombohedral	161	3.2		*n*	0.23	1269	Thin film (epitaxy)	[23]
$Zn_4B_6O_{13}$	Cubic	217	5.4		*n*	0.37	435	Bulk	[24]
Ba_2GeHfO_6	Double perovskite	225	3.94	Meta-GGA	*n*	0.45	-	-	
Ba_2PrTaO_6		225	3.74		*n*	0.69	-	-	
Ba_2PmSbO_6		225	4.28		*n*	0.38	-	-	
Ba_2SnHfO_6		225	4.36		*n*	0.37	-	-	
GeO_2	Rutile	136	4.68	HSE	*n*<br>*p*	0.27<br>1.31	288.33<br>27.98	Thin film (epitaxy)	[25]
SiO_2	Rutile	136	8.57	HSE	*p*	1.31	-	Bulk	[26]
$LiGaO_2$	Orthorhombic	33	5.1	HSE	*n*	0.4	-	Thin film (epitaxy)	[27]

References

1. E. M. Garrity, C.-W. Lee, P. Gorai, M. B. Tellekamp, A. Zakutayev, and V. Stevanović, PRX Energy **1**, 033006 (2022).

2. H. Shen, J. Wu, Z. Chen, X. Fang, J. Li, W. Li, J. Lin, F. Zhu, X. Wang, and Y. Chen, J. Phys. Chem. C **127**, 21410 (2023).

3. K. Bushick, K. A. Mengle, S. Chae, and E. Kioupakis, Appl. Phys. Lett. **117**, 182104 (2020).

4. K. Dabsamut, A. Boonchun, and W. R. L. Lambrecht, J. Phys. D. Appl. Phys. **53**, 274002 (2020).

5. P. Gorai, R. W. McKinney, N. M. Haegel, A. Zakutayev, and V. Stevanovic, Energy Environ. Sci. **12**, 3338 (2019).

6. A. Ennaciri, A. Kahn, and D. Michel, J. Less Common Met. **124**, 105 (1986).

7. Z. Sun, Y. Zhou, and M. Li, J. Mater. Res. **21**, 1443 (2006).

8. C. Hu, V. Gopal, T. Vangipuram, C. Chae, I. K. Turan, N. Hoven, W. R. L. Lambrecht, J. Hwang, Y. Ijiri, H. Zhao, and K. Kash, (2025).

9. A. N. Christensen, B. Lebech, D. Sheptyakov, and J. C. Hanson, Acta Crystallogr. B. **63**, 850 (2007).

10. T. Arlt and R. J. Angel, Phys. Chem. Miner. **27**, 719 (2000).

11. G. R. Patzke, R. Wartchow, and M. Binnewies, New Cryst. Struct. **215**, 15 (2000).

12. J. A. Speer and B. J. Cooper, Am. Mineral. **67**, 804 (1982).

13. D. Levy, A. Pavese, A. Sani, and V. Pischedda, Phys. Chem. Miner. **28**, 612 (2001).

14. K. Shiraki, T. Tsuchiya, and S. Ono, Acta Crystallogr. Sect. B **59**, 701 (2003).

15. R. D. Shannon and C. T. Prewitt, J. Inorg. Nucl. Chem. **30**, 1389 (1968).

16. H. Li, Y. Li, N. Li, Y. Zhao, H. Zhu, P. Zhu, and X. Wang, RSC Adv. **5**, 44121 (2015).

17. C.-W. Lee, K. Egbo, E. Garrity, M. Jankousky, H. Garland, A. Zakutayev, and V. Stevanović, (2025).

18. A. F. Reid, C. Li, and A. E. Ringwood, J. Solid State Chem. **20**, 219 (1977).

19. E. M. Garrity, K. Egbo, C. W. Lee, A. Zakutayev, and V. Stevanović, Chem. Mater. **35**, 9952 (2023).

20. K. Mahmood, J. Jacob, A. Rehman, A. Ali, U. Rehaman, N. Amin, S. Ikram, A. Ashfaq, and S. Hussain, Ceram. Int. **45**, 18701 (2019).

21. H. Ogawa, A. Kan, N. Ikeda, and A. Fujita, Phys. B Condens. Matter **407**, 4308 (2012).

22. D. Kovacheva and K. Petrov, Solid State Ionics **109**, 327 (1998).

23. H. Zhu, J. Liu, R. Chen, B. Feng, C. Luan, J. Ma, and H. Xiao, Vacuum **197**, 110811 (2022).

24. P. SMITH, S. GAECIA-BLANCO, and L. RIVOIR, Cryst. Mater. **119**, 375 (1964).

25. S. Chae, H. Paik, N. M. Vu, E. Kioupakis, and J. T. Heron, Appl. Phys. Lett. **117**, 072105 (2020).

26. Y. Tsuchida and T. Yagi, Nature **340**, 217 (1989).

27. S. Yasuhara, A. Nakagawa, K. Okamoto, T. Shiraishi, H. Funakubo, S. Yasui, M. Itoh, T. Tsurumi, and T. Hoshina, RSC Adv. **14**, 13900 (2024).
